# Beyond Infantile Hemangiomas: A Glimpse into Overlapping Rare Syndromes Emphasizing the Vigilant Screening for PHACE and LUMBAR Syndromes

**DOI:** 10.1155/2024/7501793

**Published:** 2024-04-18

**Authors:** Luis R. Berríos, Bianca C. Rodríguez, Mariana B. Sadurní, Karla J. Martinez, Carla M. Santiago, Natasha K. González

**Affiliations:** ^1^University of Puerto Rico, San Juan, PR, USA; ^2^University of Puerto Rico, Medical Science Campus, San Juan, PR, USA

## Abstract

Infantile hemangiomas are the most common birthmark in newborns. They are clinically diagnosed and usually self-limited. However, there are several exceptions with aggressive types of hemangiomas that can be associated with extracutaneous anomalies, such as PHACE syndrome (posterior fossa anomalies, upper body hemangiomas, arterial anomalies, cardiac anomalies, and eye anomalies) and LUMBAR syndrome (lower body hemangiomas, ulcerations/urogenital anomalies, myelopathies, bony deformities, anorectal malformations/arterial anomalies, and renal anomalies). These two syndromes, described in the literature with distinct features, have rarely been reported in the same patient. We discuss one of the few cases reported with overlapping features of the PHACE and LUMBAR syndromes that initially presented with infantile hemangiomas, as well as other nonspecific skin and systemic findings. Minimal guidance has been described due to the need for more scientific literature. Our aim is to reinforce awareness of these two syndromes and the possibility of an overlap presentation between them. Furthermore, we emphasize the need for an interdisciplinary approach with screening for all known associations to avoid missing essential components of these syndromes that can lead to significant morbidity and lifetime complications.

## 1. Introduction

Infantile hemangiomas (IH) are vascular birthmarks that affect around 3–10% of term newborns and have increased in incidence in the last 35 years [1]. This increase has been linked to an increment in premature births, low birth weight, and pregnancy complications, which are considered risk factors [2]. Other predisposing factors are Caucasian ethnicity, female sex, advanced maternal age, multiple gestation, progesterone therapy, and family history [1, 3]. While most cases are self-limited, some extensive hemangiomas can be associated with syndromes affecting other organs. Large segmental IHs in the upper body can be related to PHACE syndrome (posterior fossa anomalies, upper body hemangiomas, arterial anomalies, cardiac anomalies, and eye anomalies). If located in the lower body, IHs can be associated with LUMBAR syndrome (lower body hemangiomas, ulcerations/urogenital anomalies, myelopathies, bony deformities, anorectal malformations/arterial anomalies, and renal anomalies).

Although the precise pathogenesis remains unknown, the hypoxic uterine environment theory has received the most acceptance [1]. Evidence suggests a connection between placental hypoxia and IHs due to shared antigens, such as GLUT-1, which are crucial in hypoxic settings [4]. GLUT-1 is a precise indicator of IHs, and it has been shown to promote angiogenesis and development of IHs in cases of intrauterine hypoxia [4]. PHACE syndrome has received more coverage in the literature than LUMBAR syndrome, yet a definitive etiology remains elusive. Existing literature suggests that the anatomical features of PHACE syndrome are associated with abnormalities in the neural plate, neural crest, and adjacent cephalic mesoderm [5]. Both syndromes are uncommon in the general population, and even rarer is the occurrence of overlapping features from both syndromes in the same patient, as observed in our case. Physicians may not be aware of the syndromes, leading to a delay in diagnosis and increased morbidity for the patient. This case expands our understanding of these syndromes, thus raising awareness among physicians about the possibility of overlapping elements to promote more comprehensive screening.

## 2. Case Presentation

**Figure 1:**
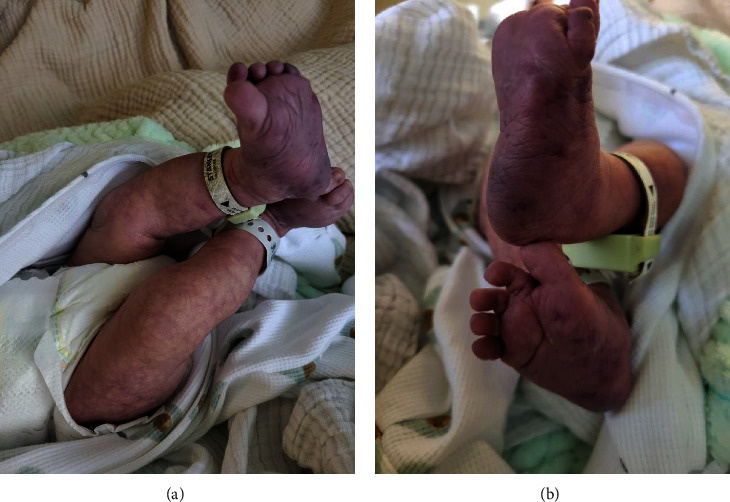
(a, b) Bluish telangiectatic pattern from the lumbar area to bilateral lower extremities associated with dark bluish-purple discoloration in the soles at one day of life.

A 1-month-old female was brought to our institution due to worsening perianal and gluteal ulcers of one-week evolution. She was born at 39 3/7 WGA via SVD without complications to a 31 y/o primigravida mother with clinically appropriate prenatal care and negative infectious serology. She had no postnatal complications. Her mother noticed diffuse bluish discoloration with a telangiectatic pattern on bilateral lower extremities and feet, extending to the lumbar area (Figures 1(a) and 1(b)). These findings were considered benign without a plan for further follow-up, and the patient was discharged home two days later.

The infant was initially doing well, but the mother noticed that the patient was less active with decreased oral intake and loose stools two days after discharge, initially attributed to feed intolerance. Given the patient's persistent hypoactivity and poor feeding, she was admitted at 16 days of age to an outside hospital with presumed neonatal sepsis. However, the patient did not meet the full criteria of SIRS (Systemic Inflammatory Response Syndrome) at admission. She was treated with empiric intravenous antibiotics for one week. Nevertheless, microbiological cultures were ultimately negative. While hospitalized, she developed a right ankle ulcer at the venipuncture site and intergluteal erythema that progressed acutely into blisters and then to nonexudative ulcers within four days after admission. The ulcers were managed with topical wound care.

**Figure 2:**
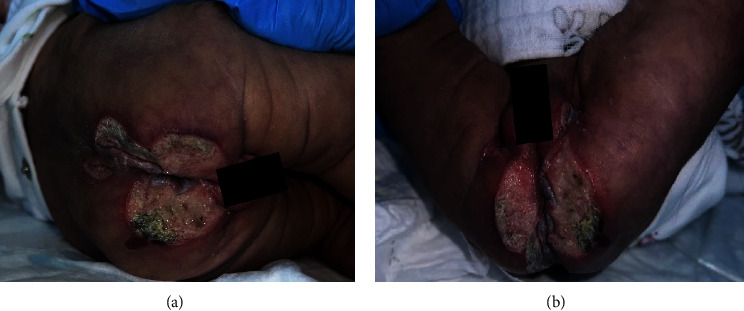
(a, b) Erythematous, purulent, and confluent ulcers at the lumbar, gluteal, and genital areas at one month of life.

**Figure 3:**
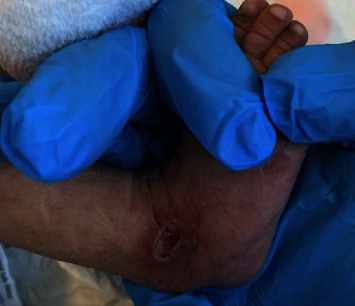
2 cm × 1 cm deep erythematous well-demarcated ulcer in the lateral right ankle at one month of life.

After completion of antibiotics, she was discharged home. The lesions showed minimal improvement after hospitalization and continued to progress, prompting further presentation to our emergency department. Upon initial evaluation, she was to be found pale, malnourished, and acutely ill. Physical examination was remarkable for mottled and reticulated lower extremities that extended to the lumbar area with confluent ulcers (Figures 2(a) and 2(b)) and a lateral right well-demarcated ankle ulcer (Figure 3). Further evaluation revealed a weak, uncoordinated sucking reflex and a bilateral positive Ortolani test with faint femoral pulses. Laboratory studies were notable for anemia (Hgb 5.8 g/dL). The coagulation studies were within normal limits.

Intravenous piperacillin-tazobactam and clindamycin were started to cover superimposed infections such as *Staphylococcus aureus* and *Pseudomonas*. She received one unit of pRBCs due to severe symptomatic anemia. A bilateral hip ultrasound confirmed dislocations with limited acetabular development. Due to faint femoral pulses, the cardiology service was consulted to assess for cardiovascular abnormalities. The transthoracic echocardiogram (TTE) revealed mild left heart dilatation, a patent foramen ovale (PFO), and a bovine aortic arch with a discrete juxta ductal coarctation of the aorta without patent ductus arteriosus (PDA). The mean gradient across the coarctation was 14 mmHg, which did not meet >20 mmHg, the intervention criteria. Appropriate abdominal aorta pulsatility without diastolic continuation of flow right below the diaphragm suggested good forward flow. The lack of proper lower extremity pulses remained enigmatic. Considering the constellation of findings, the patient was admitted and managed by an interdisciplinary team, including pediatric, dermatology, cardiology, hematology, and radiology specialists.

**Figure 4:**
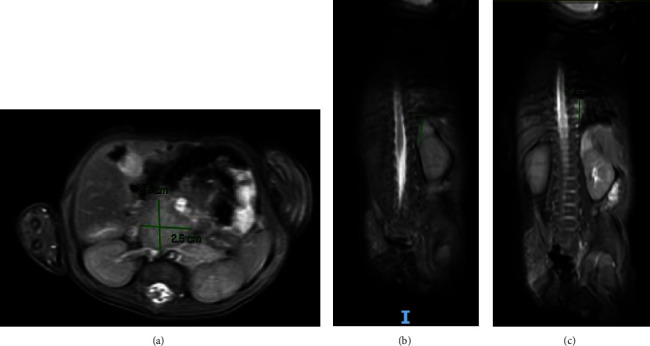
(a) Avidly enhancing rounded structure with intermediate T2 signal and mild restricted diffusion seen in the retroperitoneum between the aorta and SMA, and inferomedial to the expected position of the pancreatic head, anterior to the IVC vein measuring approximately 2.6 cm AP × 2.5 cm transverse × 2.6 cm. (b) Ill-defined enhancing structure, with marked T2 hyperintensity and no restricted diffusion in the left adrenal region measuring approximately 1.5 × 2.3 by 2.3 cm, abutting the left renal upper pole, without a definite fat plain between them. (c) Left paraspinal mass (grossly at the level of T8–T10) with enhancement, high T2 signal, and mild restricted diffusion approximately 6 mm AP × 2.1 cm TR × 2 cm long. There is mild extension to the left T7-T8 neural foramen without appreciable expansion.

**Figure 5:**
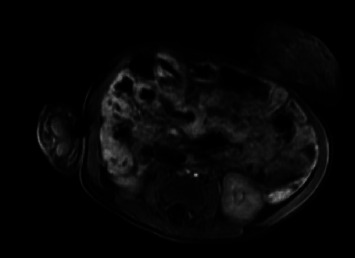
Merely diffuse significant bowel wall thickening and hyperenhancement throughout most of the small and large bowel. No free air identified.

A punch biopsy of the gluteal ulcers revealed non-inflammatory spongiotic dermatitis with telangiectatic vascular channels in the superficial dermis positive for GLUT-1, CD-31, and CD-34 markers, which are specific for IH. The physical findings and the pathological report were consistent with an extensive IH with minimal growth (IH-MG) that ulcerated. An abdominopelvic MRI was performed to assess common extracutaneous IH locations and was remarkable for an ill-defined posterior perirectal region that was correlated with a gluteal hemangioma and enhancing lesions at the retroperitoneum, left adrenal, and left paraspinal area that were compatible with additional IHs (Figures 4(a)–4(c)). The liver had a normal size and morphology. No suspicious hepatic lesions were observed. This MRI also revealed nearly diffuse thickening and hyperenhancement of the bowel wall, suggesting infiltrating intestinal hemangiomatosis (Figure 5). The CTA demonstrated a discrepancy between the distal abdominal aorta and iliac vessels of the proximal (6 mm) and distal aorta (3 mm), identifying a vascular anomaly. After extensive workup, the team suspected LUMBAR syndrome and TTE suggested an overlap with features of the upper counterpart, PHACE syndrome. We performed a spinal ultrasound, which revealed no evidence of dysraphism. A brain MRI/MRA was performed to evaluate possible anomalies related to PHACE syndrome, with unremarkable findings. The oncology team was consulted concerning the hyperintense left adrenal mass, suggesting neuroblastoma. However, urine catecholamine levels were found to be within the normal range. Thyroid stimulating hormone and thyroxine hormone tests were not performed.

For proper management of IH, the patient began with intravenous Propranolol 1 mg/kg/day every 12 hours and slowly titrated to a maximum dose of 2 mg/kg/day every 12 hours for three months. After three months of Propranolol and wound care, the ulcers healed appropriately. Since the patient's ulcers dramatically improved, the parents expressed their interest in continuing care in their home state, as they were not native to Puerto Rico. A follow-up abdominopelvic MRI was performed to evaluate the progression of internal IHs. Imaging revealed an interval improvement of the hyperintense lesions previously mentioned. Given the patient's adequate oral intake tolerance and appropriate weight gain, she was discharged to continue oral Propranolol, with emphasis on the importance of follow up and continuation of care in her home state.

## 3. Discussion

### 3.1. Overview

We describe a rare case where nonspecific skin and systemic findings were initially dismissed as benign and eventually led to the identification of LUMBAR syndrome characteristics with overlapping PHACE syndrome criteria. As described earlier, PHACE syndrome has been studied more, leading to a criterion for its diagnosis. According to the most recent expert consensus for its diagnosis, management, and complications, the criteria for the definitive PHACE syndrome are based on the presence of a facial or scalp hemangioma >5 cm plus one primary criterion [6]. Those who present with large segmental hemangiomas in the neck, upper trunk, or upper extremities that also meet at least two major criteria can also be diagnosed with definitive PHACE [6]. Patients who do not complete the definitive diagnosis or do not have IH in the upper body but who present with part of the spectrum of PHACE can meet the criteria for a possible diagnosis.

In contrast, there is currently no officially established consensus-based diagnosis for LUMBAR syndrome. Instead, it can be identified based on the clinical and imaging findings. Early screening is critical, given that LUMBAR syndrome is easily misdiagnosed as cutis marmorata telangiectasia congenita, reticulate capillary malformation, or even as a diaper rash [7, 8]. This explains why our patient was initially discharged home without further intervention. Thus, it is critical to guide general pediatricians toward identifying high-risk skin lesions that may merit referral and management.

### 3.2. Characteristics of the LUMBAR Syndrome and Overlapping Features

Our patient presented at birth with the classical morphology associated with segmental IH with minimal growth––the most common type associated with segmental hemangiomas of LUMBAR syndrome [9]. Previous publications have found that large hemangiomas in the extremities are more likely related to extracutaneous anomalies such as arterial malformations and limb underdevelopment [10]. Among previously reported cases, 16% exhibited arterial anomalies, with the iliac and superficial femoral arteries being the most implicated [11]. Similarly, our patient developed bilateral hip dysplasia with limited acetabular development, possibly attributed to reduced perfusion, as indicated by the decreased caliber at the lower abdominal and bilateral iliac branches in CTA.

Besides vascular abnormalities, LUMBAR syndrome commonly presents with myelopathy, observed in approximately 80% of cases, with the tethered cord being the most common finding [9]. According to scientific literature, all patients with segmental IH of any lumbosacral or perineal region under three months should undergo ultrasound scanning of the spine, abdomen, and pelvis with color Doppler [9]. Compared to MRI, ultrasound is sometimes preferred in younger infants, as it facilitates the detection of associated defects without sedation. However, 74% of pediatric neurosurgeons recommend MRI as the best imaging modality when surgery is necessary. Our patient did not present with urogenital, anorectal, or renal anomalies, which can be seen in 15%, 28.5%, and 17.6% of patients with LUMBAR syndrome, respectively. Nevertheless, long-term complications and disfigurement could be observed in this patient once the ulcerated hemangiomas start to involute and create scars of comparable size.

In a retrospective study, Shayegan et al. evaluated 22 cases of patients with overlapping features. Among these cases, three showed lower body IH with cardiac and arterial PHACE-associated congenital abnormalities and five with upper body IH with LUMBAR-associated congenital anomalies [12]. Based on the TTE findings, our patient met two major criteria in the PHACE spectrum: a bovine arch (arterial anomalies) and a coarctation of the aorta (cardiovascular anomalies). Along with no physical finding of hemangiomas in the upper body, we suggest that our patient fulfills the criteria not only for LUMBAR but also for possible PHACE syndrome. These findings, unlikely to be coincidental, may represent a continuum spectrum of these syndromes that deserve further investigation.

### 3.3. Complications and Management of IH

IH may serve as an initial indication for structural syndromes such as PHACE and LUMBAR. Evidence suggests that PHACE is associated with 30% of segmental IHs on the face, scalp, and neck. When large (>2.5 cm) midline or segmental IH is identified in the lumbosacral/perineal location or lower extremities combined with anatomic anomalies are identified, LUMBAR syndrome must be considered [11]. Early identification of ulceration is crucial, as it is the most common complication, carrying an added risk of secondary infection and, notably, excruciating pain—the most prevalent problem associated with ulceration. Ulceration is more likely to occur when the IH has extensive or minimal growth, like in our case [7]. Other high-risk regions include the neck, face, lips, extremities, and intertriginous areas due to friction and maceration. Moreover, IH >2 cm (infants aged ≥3 months) or >1 cm (infants aged <3 months) are more likely to ulcerate [3]. In case of ulceration, infants can be treated with Propranolol, pain medications, and wound dressing. Other complications may include obstructive airway if IHs develop in the neck, functional impairment of the eyes or mouth, and possible disfigurement.

### 3.4. Awareness and Screening Strategies

Prompt diagnosis and intervention decrease the risk of serious complications. Raising awareness among general pediatricians (GPs) and parents about the diverse characteristics of IH is crucial for effective management. Parents should be educated on actively monitoring the lesions and communicating any concerns [3]. Incorporating a skin check during the first six weeks of life, along with a comprehensive evaluation of the abdomen, genitals, and neurological status, can facilitate timely detection [3]. To assist general pediatricians, Léauté-Labrèze et al. published an easy-to-use validated screening tool (IHReS) with a sensitivity of ∼97% to identify patients who require a prompt referral to a specialist [13]. This free tool can be easily found at https://www.ihscoring.com/.

## 4. Conclusion

We present a rare case of a 1-month-old patient with nonspecific symptoms, including failure to thrive and poor feeding associated with lumbosacral ulcers. The diagnosis revealed overlapping features of both LUMBAR and PHACE syndromes. This case highlights the rare phenotypic spectrum of these poorly understood syndromes and suggests that there could be a common etiology yet to be found. Even though new therapies can emerge, this type of case can help researchers understand and develop pathogenesis-directed therapies.

Given that there is no expert consensus on a diagnostic criterion for LUMBAR, a high level of suspicion is crucial for GPs, dermatologists, and vascular physicians. GPs play a pivotal role in the management of these cases, and we recommend that they utilize standard tools for IH screening while also considering potential overlapping features. This approach ensures a thorough assessment and management to prevent serious complications.

## Abbreviations

WGA: Week gestational age
SVD: Spontaneous vaginal delivery
pRBC: Pure red blood cells
U/S: Ultrasound
GLUT-1: Glucose transporter-1
CD-31, 34: Cluster of differentiation-31, 34
MRI: Magnetic resonance imaging
CTA: Computer tomography angiography
MRA: Magnetic resonance angiography
IHReS: Infantile hemangiomas referral score.
